# Utility of Conventional but Late Pulmonary Artery Banding in Complex Cyanotic Congenital Heart Disease in a Toddler - A Single Case Scenario

**DOI:** 10.7759/cureus.35452

**Published:** 2023-02-25

**Authors:** Vishal V Bhende, Tanishq S Sharma, Ashwin S Sharma, Krishnan G Subramaniam, Amit Kumar, Krutika R Tandon, Dhruva Sharma, Gurpreet Panesar, Kunal Soni, Kartik B Dhami, Sohilkhan R Pathan, Nirja Patel, Hardil P Majmudar

**Affiliations:** 1 Pediatric Cardiac Surgery, Bhanubhai and Madhuben Patel Cardiac Centre, Bhaikaka University, Karamsad, IND; 2 Community Medicine, Sal Institute of Medical Sciences, Ahmedabad, IND; 3 Medicine, Gujarat Cancer Society Medical College, Hospital and Research Centre, Ahmedabad, IND; 4 Pediatric Cardiac Surgery, Sri Padmavathi Children's Heart Center, Tirupati, IND; 5 Pediatric Cardiac Intensive Care, Bhanubhai and Madhuben Patel Cardiac Centre, Bhaikaka University, Karamsad, IND; 6 Pediatrics, Pramukhswami Medical College, Bhaikaka University, Karamsad, IND; 7 Cardiothoracic and Vascular Surgery, Sawai Man Singh (SMS) Medical College and Hospital, Jaipur, IND; 8 Cardiac Anesthesiology, Bhanubhai and Madhuben Patel Cardiac Centre, Bhaikaka University, Karamsad, IND; 9 Clinical Research Services, Bhanubhai and Madhuben Patel Cardiac Centre, Bhaikaka University, Karamsad, IND

**Keywords:** pulmonary venous hypertension, unrestricted pulmonary blood flow, pulmonary vascular resistance, pulmonary vascular disease, single ventricle physiology, late pulmonary artery band

## Abstract

Newborns with untreated single ventricles develop pulmonary vascular diseases early in their lives. At that age, during the first eight weeks after birth, clinicians perform pulmonary artery (PA) banding to reduce the blood flow to the lung, decreasing the likelihood of future high vascular resistance or pressure. PA banding is also considered an initial stage in the process of single ventricle palliation procedures. We report a case of a 16-month-old toddler (7 kg) with room air saturation of 82%, diagnosed with tricuspid valve atresia, large atrial and ventricular septal defect, and hypoplastic right ventricle with severe pulmonary arterial hypertension. The baby underwent a successful surgical procedure of PA banding and was discharged after 13 days of hospital stay with a room air saturation of 89%. This case highlighted the benefit of PA banding beyond the stipulated period.

## Introduction

In 1952, Muller and Dammann introduced pulmonary artery (PA) banding as a palliative surgical procedure for patients with congenital heart abnormalities that involve a left-right shunt [high pulmonary blood flow (PBF). The procedure aims to prevent irreversible changes in the pulmonary vascular bed while the patient is awaiting definitive surgery to correct the congenital cardiac defect [1]. In the case of single-ventricle (SV), palliative management consists of multiple surgical stages to relieve the obstruction in front of the systemic cardiac output and guarantee unrestricted PBF. PA banding is the first surgical stage in the absence of systemic flow obstruction [2-6]. 

PA banding is used for a few types of ventricular septal defects and SVs. Moreover, it is usually done within the first eight weeks after birth, while it is not recommended after 12 weeks [7]. Our study reports the clinical improvement and assesses the patient's eligibility for bidirectional Glenn (BDG) shunt after the late PA band surgery (second-stage surgery).

## Case presentation

The Institutional Ethics Committee (IEC-2) at H.M. Patel Centre for Medical Care and Education, Anand, Gujarat (Approval No. IEC/BU/2022/Cr.19/76/2022, dated 02.06.2022) approved this study. The patient's parents gave their consent to use the medical data of their child.

Presentation

**Figure 1:**
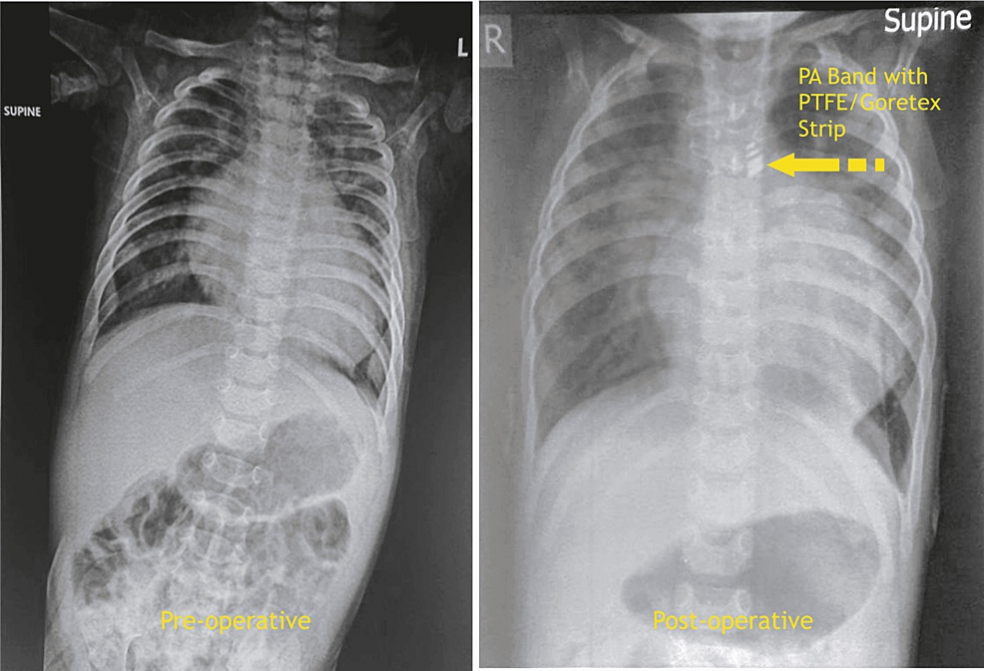
Preoperative and postoperative chest X-ray of a 16-month-old patient with pulmonary artery band in situ PA - pulmonary artery, PTFE - polytetrafluoroethylene

A 16-month-old girl (7 kg) presented to our pediatric cardiac outpatient clinic with respiratory distress, cyanosis, and room air saturation (SpO_2_) of 82 % in the four limbs. The patient was born at term after a low-segment Caesarean section with a birth weight of 3.1 kg. and cried immediately after birth (CIAB). On chest X-ray, we notice an increase in pulmonary vascularity besides clear cardiomegaly (Figure 1).

**Figure 2:**
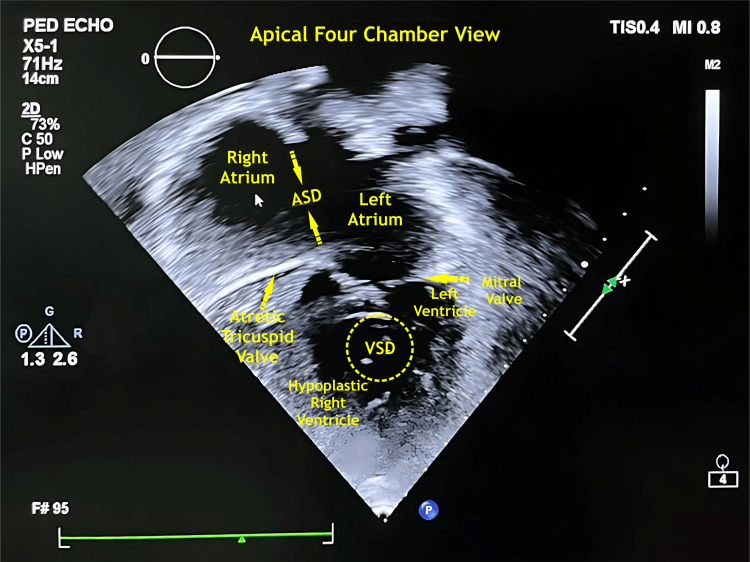
2D echocardiography apical four-chamber view ASD - atrial septal defect, VSD - ventricular septal defect

Her 2D echocardiography revealed tricuspid valve atresia (type-I c), large atrial septal defect (ASD) with right-left shunt, large ventricular septal defect (VSD) with left-right shunt, hypoplastic right ventricle, dominant left ventricle, and severe pulmonary arterial hypertension. Before the PA banding procedure, the toddler underwent complete preoperative clinical examinations, including a cardiac and neurological examination. Besides routine laboratory investigations, qualitative molecular analyses were negative for SARS-CoV2 (COVID-19) (Figure 2).

Technique of pulmonary artery banding

**Figure 3:**
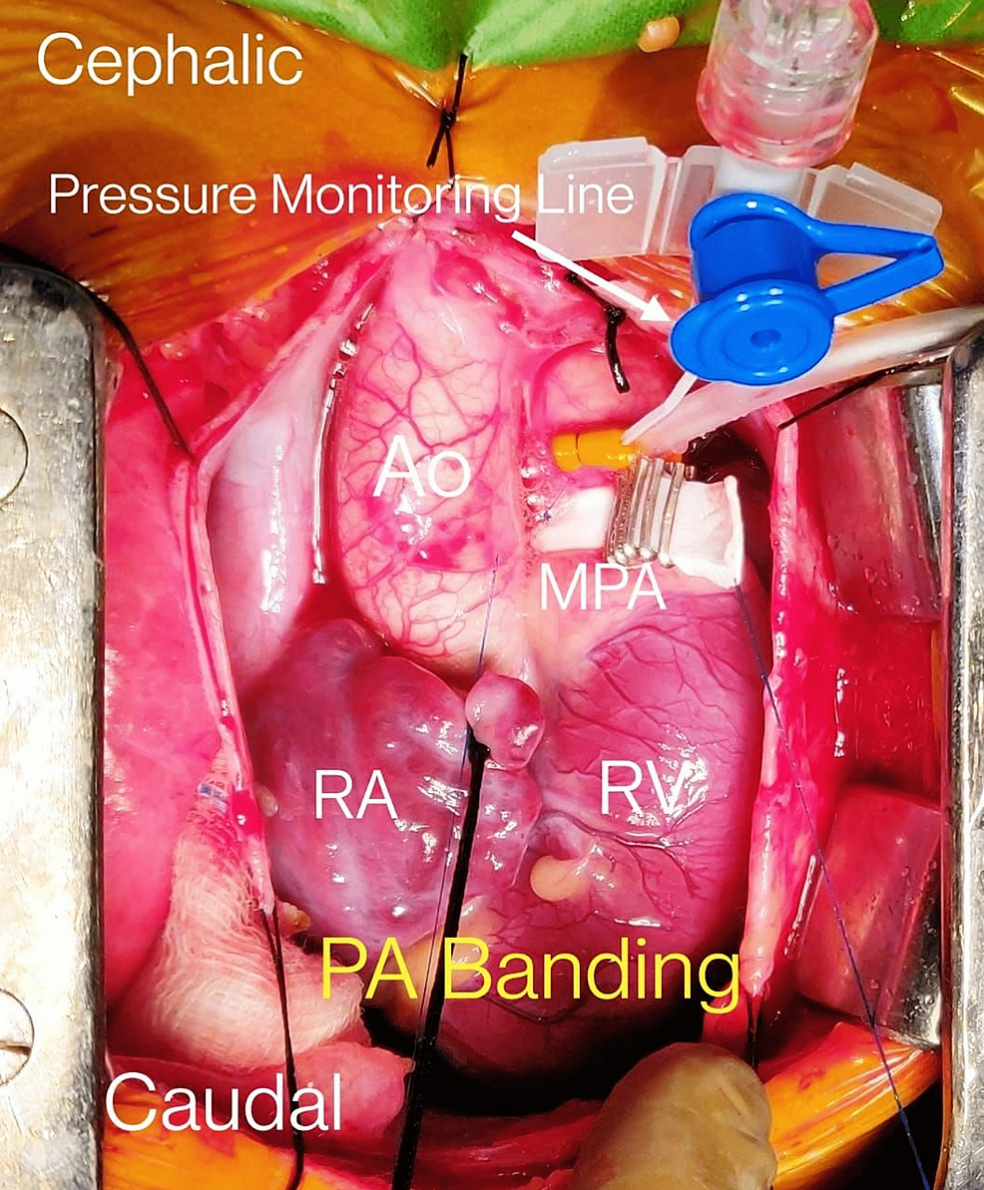
Intraoperative demonstration of PA banding procedure in a 16-month-old toddler PA - pulmonary artery, Ao - aorta, MPA - main pulmonary artery, RA - right atrium, RV - right centricle

Through a median sternotomy entry, we carefully performed a minimal dissection between the aorta and the main PA. GORE-TEX strip (WL Gore and Associates, Flagstaff, Arizona) was used for banding. The width was 3-5 mm, while the thickness equaled 0.6 mm. Subsequently, the band was placed without distorting the nearby PA branches. However, we fixed it with a 5/0 or 6/0 polypropylene suture to the adventitia of the main PA. There was minimal blood loss, and the whole procedure took up to two hours (Figure 3, Video 1).

**Video 1:** Pulmonary artery banding - 3D medical animation

Formula Employed for Band Circumference

The initial assessment of the band diameter was conducted as Trusler (1972) previously described. Accordingly, the diameter for infants with simple defects without intra-cardiac bi-directional mixing disorders is (20 mm + 1 mm)/kg. At the same time, it is (24 mm + 1 mm)/kg in the presence of bi-directional mixing disorders.

Further readjustment by tightening the band was performed to fulfill a distal PA pressure below 50% compared to a systemic one, and SpO_2_= 80% when FiO2 is nearly equal to 21%. Any new onset arrhythmia was considered a non-tolerance for the band. Echocardiography was performed to measure the supravalvar diameter and pulmonary valve diameter during the surgery; the accepted reduction in supravalvar diameter was approximately 50% (Video 2).

**Video 2:** Utility of conventional but late pulmonary artery banding in complex cyanotic CHD CHD - cyanotic heart disease

After an uneventful operation, the patient was transferred to the cardiac surgical intensive care unit, where she was monitored on mechanical ventilation and milrinone support. Subsequently, she was put on peritoneal dialysis due to mild low cardiac output syndrome. On the day of the operation, i.e., postoperative day (POD) 0, the baby had low cardiac output with a drop in urine output and a rise in potassium levels. To improve her renal function, peritoneal dialysis was initiated on POD 0 and continued till POD 5 (Day 5 after surgery) when the child was extubated (after 122 hours of ventilatory time). It was discontinued as the edema settled and renal function improved. No pulmonary edema was noted. The patient was gradually introduced to a liquid diet and then full meals; finally, she was discharged in stable condition. At the time of discharge, her SpO_2_ was 89% on room air with a peak gradient across a band of approximately 54 mm Hg (Table 1). A clinical improvement was observed during the postoperative follow-up period (three months), including weight gain and fewer respiratory tract infection episodes.

**Table 1:** PA banding parameters of a 16-month-old after PA banding operation ABP - arterial blood pressures, PAP - pulmonary artery pressures, PA - pulmonary artery

Parameters	Pre-band	Post-band
Heart rate	126 min	142/min.
SpO_2_	100% on FiO_2_ 60%	97% on FiO_2_ 50%
PAP	42/17 (30) mm Hg	37/17 (25) mm Hg
ABP	82/40 (54) mm Hg	95/44 (63) mm Hg

**Figure 4:**
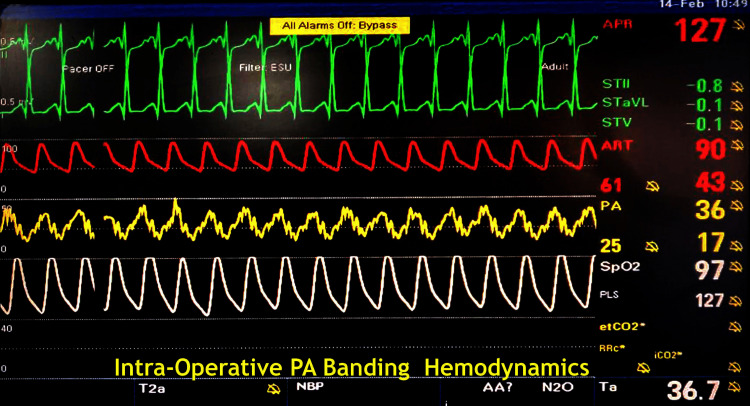
Intraoperative demonstration of PA banding gradient (in yellow tracing) procedure in a 16-month-old toddler PA - pulmonary artery

**Figure 5:**
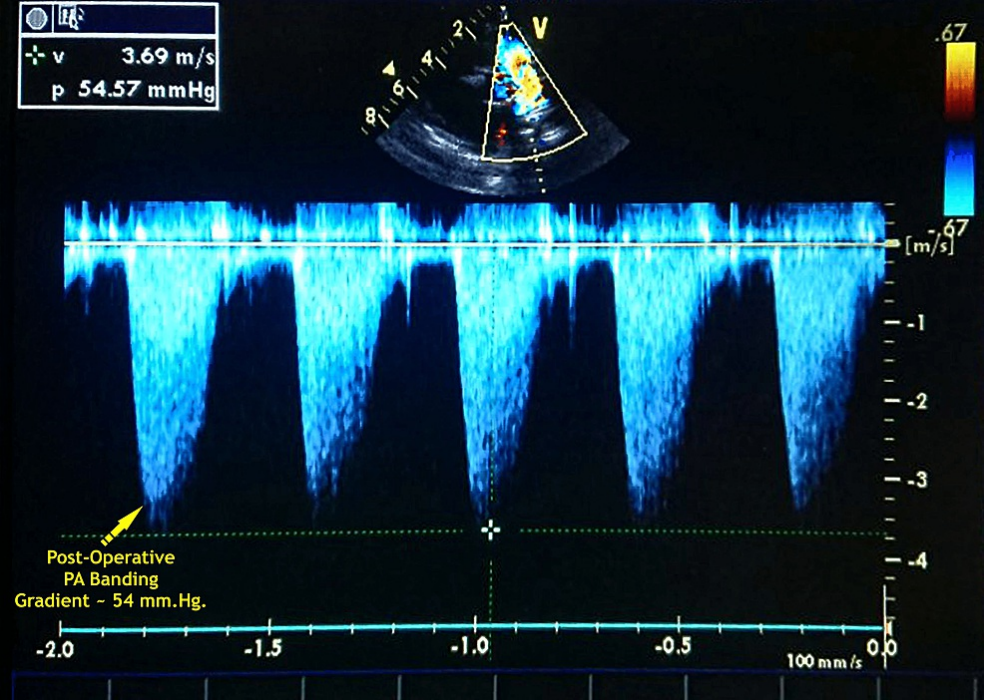
Postoperative demonstration of PA banding gradient procedure of 54 mm Hg in a 16-month-old toddler PA - pulmonary artery

Pulmonary artery band gradient = ABP-PAP (both systolic) = 95−37=58 mm Hg. The PA band was fixed at 26.5 cm circumference approximately on the Goretex/ polytetrafluoroethylene (PTFE) strip (Figures 4, 5)

## Discussion

SV with pulmonary stenosis is usually diagnosed early due to its unique signs. Conversely, SV with high blood flow in the pulmonary vascular bed lacks clinical manifestations such as clear cyanosis and harsh murmur, making the diagnosis difficult. Therefore, early diagnosis is essential for SV patients with unrestricted PBF as the suitable age for surgical repair is four to eight weeks; however, some experts consider 12 weeks suitable as well [7]. Though, the diagnosis is still challenging because of late presentation as they remain asymptomatic during the first weeks after birth, which may prevent them from PA banding. The issue is even more critical in developing countries due to limited resources such as fetal echocardiography and a robust screening program as well as mandatory pulse oximetry recording for neonates [8-10].

However, nearly 75% of patients with SV do not survive within the first three years of their life [11]. In the case of proven associated unrestricted PBF, the survival rate becomes only 10% for children above three years old [12, 13].

Early mortality causes include recurrent respiratory infections, refractory heart failure, and severe growth failure. However, even if some patients survive beyond three years, they usually develop early-onset pulmonary vascular disease (PVD) much quicker than the standard left-to-right shunts. Furthermore, these complex defects have a poor survival rate of nearly two decades compared to standard Eisenmenger patients [14].

Through this case, we tried to determine whether late PA banding can halt the disease progression for the patient to become eligible for BDG (second-stage palliation). In the case we reported, a clinical improvement was observed during the postoperative follow-up period (three months), including weight gain and fewer respiratory tract infection episodes. Persistent weight gain is an essential parameter that reflects infant well-being.

SV defect may come with another defect, such as cor triatriatum and supramitral ring, in addition to mitral valve stenosis, that may obstruct pulmonary venous return, leading to pulmonary venous hypertension (PVH), which worsens the situation of children above one years old. PVH also participates in the pathology of pulmonary arterial hypertension (PAH) passively by elevating pulmonary venous pressures and actively by causing pulmonary arteriolar vasoconstriction [15,16]. Our patient did not show any features of PVH. However, atrial septectomy procedures with a PA band reduce the risk of PAH and PVH development.

Previous studies have investigated PA banding outcomes in SV patients. However, Sasikumar et al. are the only ones who examined the outcome of late PA banding. He enrolled 32 patients above eight years [17]. The cut-off age was 28 days (34% above six months), with a few below three months. As additional procedures, some patients required arch repair, Damus-Kaye-Stansel repair, VSD enlargement, etc. However, a mortality rate of 15.5% was seen in their study.

The current consensus defines the criteria for patients who deserve a chance of PA banding, even at a late age, within the first six months of life. That includes SpO_2_ above 85% in room air and proved high PBF (clinically, radiologically, and by echocardiographic tools), which is irrespective of the presentation age. PA band may hinder PVD progression and reduce the number of eligible patients for SV palliation. Patients who adhere to the Fontan pathway unequivocally show better long-term outcomes than those who do not.

## Conclusions

Managing SV with unrestrictive PBF is challenging for infants in the developing world. Even when performed beyond the stipulated period, the PA band might improve patients' quality of life. Patients who undergo late PA bands may also progress to SV palliation and get better outcomes even if they do not fit into such a category. Surgeons should always keep in mind the risk-benefit ratio before going forward with this procedure. However, definite conclusions cannot be drawn at this point in time until more research data is available in this subset.
